# Neck Disability Index Detects Higher Neck-Related Disability Levels among Physiotherapists and Family Medicine Specialists than among Dentists

**DOI:** 10.3390/healthcare11040581

**Published:** 2023-02-15

**Authors:** Jure Aljinović, Blaž Barun, Benjamin Benzon, Ivanka Marinović, Ante Aljinović, Ana Poljičanin

**Affiliations:** 1 Institute of Physical Medicine and Rehabilitation with Rheumatology, University Hospital of Split, Šoltanska 1, 21000 Split, Croatia; 2 University Department of Health Studies, University of Split, 21000 Split, Croatia; 3 Departments of Anatomy, Histology and Embryology, School of Medicine, University of Split, 21000 Split, Croatia; 4 School of Medicine, University of Split, 21000 Split, Croatia

**Keywords:** work related disability, dentist, physiotherapist, family medicine, neck disability

## Abstract

Background: Healthcare workers who are in physical contact with patients are prone to work-related musculoskeletal disorders (WMSDs). Much is known about the prevalence of neck pain, but the extent of disability associated with neck pain among physical therapists (PTs), dentists, and family medicine specialists (FMs) is unknown. Methods: The prevalence of neck pain and Neck Disability Index (NDI) data were collected from 239 PTs, 103 FMs, 113 dentists, and 112 controls from June to August 2022. Results: The highest prevalence of neck pain was found in FMs (58.3%), followed by dentists (50.4%), PTs (48.5%) and controls (34.8%). The NDI% in PTs and FMs had higher values than controls: 14.6 ± 12.4, *p* = 0.02 for PTs, 14.9 ± 12.4, *p* = 0.01 for FMs vs. 10.1 ± 10.1 controls. The dentist group did not differ from controls (11.9 ± 10.2, *p* = 0.13). Mild, moderate, or severe forms of disability were more common in medical professionals than in controls (44.2%, 9.5%, and 1.5% vs. 37.5%, 7%, and 0%). Dentists were the youngest group with high functionality and the lowest degree of disability, comparable to the control population. Gender or age had no effect on NDI scores in this population. FMs, who represented the oldest group, showed age dependency (eleven years older in higher disability groups). Gender had no effect on NDI. In PTs, females predominated in all disability categories and PTs became five years older with increasing disability level. Conclusion: By using NDI in assessing neck-related WMSDs, we can detect medical professionals prone to more serious disability and potentially plan preventive actions.

## 1. Introduction

Work-related musculoskeletal disorders (WMSD) describe a wide range of diseases or disorders that result in pain paired with or without functional impairment. The most common aetiology of WMSD is simple mechanics (physical activity involving repetitive tasks, high force manual techniques, work techniques that are done in awkward positioning, and prolonged static muscular constrained postures), followed by inadequate working tools or ergonomics (use of vibrating tools, insufficient training in ergonomics during preclinical and clinical courses at universities, inappropriate workplace/facilities design) sometimes combined with psychological factors that exert physical and mental stress on medical professionals such as increase of workload, time pressure, little autonomy over work, monotonous work and lack of support from colleagues or superiors [1,2,3,4,5,6,7,8,9,10,11,12,13,14,15,16,17,18,19,20,21,22,23,24,25,26].

Healthcare workers who are in physical contact with patients are subjectable to WMSDs due to the requirements of their jobs. Forced body positions maintained throughout the day can lead to chronic pain syndromes. Among dentists, neck pain is the first cause of WMSDs, according to a 2022 systematic review by Halkai et al. [1], and the second leading cause of WMSDs in physiotherapists (PTs), as described in a narrative review by Milhem et al., in 2016. [2].

Among PTs, a lifetime prevalence of neck pain of 12–59% has been reported [3,4,5,6] with similar numbers of 12-month prevalence of 18–57% [3,6,7,8,9,10,11]. Female gender is associated with a higher incidence of neck pain as a result of WMSDs [3,9,10,12], while only Cromie et al. reported a higher incidence in male PTs [11]. An association between neck pain and PTs age has not been demonstrated, in con trast to low back pain, which is more common at younger ages [10].

The lifetime prevalence of neck pain is even higher in dentists than in PTs, ranging from 15 to 91% [13,14,15,16,17,18,19]. In Croatia, a lifetime prevalence of upper back pain was reported to be 78%, but upper back pain was not divided into neck and thoracic pain, which may mean that the percentage of neck pain is smaller than the total [20].

Data on WMSDs in primary care among specialists such as family medicine specialists (FMs), often referred to as general practitioners, are scarce, but a paper by Rambabu et al. described musculoskeletal pain occurring in 61% of dentists vs. 20% of physicians [21], while Kitzmann et al. described a higher prevalence of neck pain among ophthalmologists than FMs (46% vs. 21%) [22].

Although much is known about the prevalence of neck pain, very little is known about the degree of disability it causes in healthcare workers. The most common method for researching this topic is functional indices. Neck pain-related disability is frequently measured by the Neck Disability Index (NDI). NDI in Croatian language was previously validated in whiplash injury patients, physiotherapists and control population. Reliability testing showed strong correlation in test-retest experiment and in item-total score correlation test in all ten categories of NDI for all groups [24]. Validity testing showed positive correlation between NDI and pain VAS [24]. Psychometric characteristics of NDI-CRO were analysed by correlation of final score with age, gender and coexistence of depression through Patient Health Questionnaire 9 (PHQ-9) [24]. No correlation was found between age, gender and final NDI score. Positive correlation between PHQ-9 and NDI score was detected in all three groups emphasizing effect of depression on higher NDI score

To the best of our knowledge, there are no data in the literature on functional indices for neck degree disability in dentists and FMs.

WMSDs cause substantial economic burden to individuals and the community as the treatment expenditures are relatively high [1,2]. This paper hopefully represents one of the many to follow evidence-based knowledge about at-risk populations and the need to implement or strengthen the preventive care. The objective of this paper is to determine the degree of disability among PTs, dentists, and FMs in Croatia who reported neck pain at the time of the study and to detect at-risk medical professionals who would benefit from preventive care programs.

Our hypothesis is that dentists and PTs will have higher degrees of disability than FMs and the control population. We based our assumption on the fact that dentists and PTs are among the health care professionals who have the highest prevalence of WMSDs [25] while FMs experience WMSDs less frequently and we believe that disability degrees should corelate to this data.

## 2. Materials and Methods

This cross-sectional study was conducted from June to August 2022 in Croatia. Croatia is divided in the continental part represented by participants from Vukovar County and the Mediterranean part represented by people from Dalmatia County.

### Patient and Public Involvement Statement

This study was approved by the Ethics committee of the Department for Health Studies, the University of Split (No. 029-03/22-08/01). Informed consent was obtained from all the participants. Eligible participants were approached at their place of work either by the authors alone or by a person selected by the institution where they work, after written or verbal approval by the ethics committee of that institution. All NDIs were delivered in paper form. For the participants recruitment purposes the authors contacted all Clinical Hospital Centres, General Hospitals, and Community Health Centres where PTs, dentists, and FMs work, as well as some of the private practises in the regions studied. There were no exclusion criteria.

In the enrolment process 73% of approached FMs decided to complete the questionnaire (103/148), 80% of the PTs (239/297) and 75% of dentists (113/149). According to the data publicly available with medical chambers of different professions in Croatia: the data from 4.6% of all FMs working in Croatia (103/2223), 12.1% of all PTs (239/1975) and 2.2% of dentists (113/5066) were obtained.

Main reasons for declining participation in this study were: I don’t have time, I’m not interested, I have a lot of patients waiting (Supplementary Figure S1).

The control group consisted of 112 randomly selected individuals from the everyday population. All participants in the medical professional groups spoke Croatian and were between 27 and 75 years old, while the age range in the control group was from 19 to 71 years.

Data on age, gender and occupation were obtained followed by NDI questionnaire. No other demografic or work-related data were collected due to the time limitation since participants were approached at their place of work.

For measuring neck pain-related disability Croatian version of Neck Disability Index (NDI) was chosen.

The NDI is a condition-specific questionnaire that assesses the degree of disability caused by neck pain and/or restricted neck movement in everyday activities (self-care, lifting, reading, occurrence of headaches, concentration, driving, sleeping, occupational, and recreational activities) [23].

The total NDI score was divided by the maximum possible score and the index percentage was calculated (NDI%). Scores of 0–8% were considered no disability, 10–28% mild disability, 30–48% moderate disability, 50–68% severe disability, and 70–100% complete disability. If one or two answers remain unanswered, the NDI is still acceptable, with a maximum value of 45 or 40 divided by the number of questions answered [23]. All patients who gave an answer other than “I have no neck pain” to the first question of the NDI were classified as positive for neck pain and counted in the point prevalence. The translated Croatian version of NDI is freely available for academic usage at: https://eprovide.mapi-trust.org/instruments/neck-disability-index#languages.

All statistical analyses were performed using Past 4.11 software (Natural History Museum, University of Oslo, Norway). Age was described as median and interquartile range (IQR) and compared with the Kolmogorov-Smirnov test because of the bimodal distribution of the data. NDI% was described as mean ± SD. Scores for each NDI category were compared between groups using multiple linear regression with dummy variables representing PTs, FMs, dentists, and control groups. Control group was used as a referent category. Statistical significance was set at α = 0.05.

## 3. Results

PTs and the control group are age matched (43, IQR 34–54 vs. 43, IQR 29–58, *p* = 0.07), while dentists were younger (42, IQR 30.5–48.5, *p* = 0.002) and FMs were older (49, IQR 36–59 vs. 43, IQR 29–58, *p* = 0.02) than the control group. Baseline demographics are shown in Table 1.

There is a substantial female predominance in medical professional groups: 81.5% in FMs, 74.5% in PTs, and 62.8% in dentists. The control group consisted of 50.9% men and 49.1% women (Table 1).

The highest prevalence of neck pain was found in the FMs group, 58.3%, followed by 50.4% in dentists, 48.5% in PTs, and 34.8% in controls.

The NDI% was calculated for each group, PTs (n = 239) and FMs (n = 103) had higher values than the control population (n = 112): 14.6 ± 12.4, *p* = 0.02 for PTs, 14.9 ± 12.4, *p* = 0.01 for FMs vs. 10.1 ± 10.1 controls. The dentist group did not differ from the control group in NDI% (11.9 ± 10.2, *p* = 0.13).

**Figure 1:**
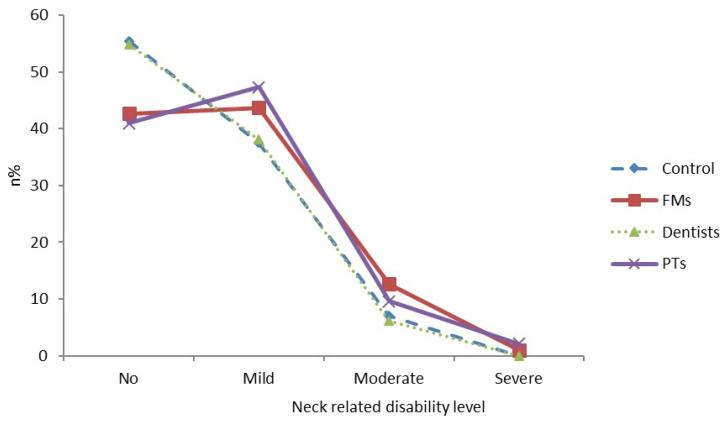
Level of neck pain related disability in different study groups obtained by NDI (Neck Disability Index). n%—percentage of total participants in group, FMs—family medicine specialists, PTs—physiotherapists, control—control population, dentists.

A further breakdown of the NDI% scores into degrees of disability can be seen in Figure 1. Among dentists, there is a sharp decrease in the line connecting the disability levels from 55% with no disability to 38% with mild disability and 6% with moderate disability. This line is almost identical to that of the control population (Figure 1).

In contrast, the line connecting no disability and mild disability stagnated or even slightly increased for both FMs and PTs (42% vs. 43% for family medicine and 41% vs. 47% for PTs, respectively, Figure 1). Moderate disability was also a significant problem in these groups, at 10% for PTs and 12% for FMs.

Females had a higher NDI% than males in the control population (13.1 ± 11.7 vs. 7.1 ± 7.4, *p* = 0.02) and in PTs (16.2 ± 13.1 vs. 10 ± 9.1, *p* = 0.007), while FMs (15.4 ± 12.4 vs. 13 ± 12.8, *p* = 0.6) and dentists (11.5 ± 10.4 vs. 10.6 ± 10.1, *p* = 0.08) had no gender differences. Further breakdown of gender and age of participants into different groups of neck-related disabilities is shown in Table 2. Neck-related disabilities are more common in medical professionals in mild, moderate, or severe forms than in the control population (44.2%, 9.5%, and 1.5% vs. 37.5%, 7%, and 0%, respectively).

For mild disability, females predominate in the control population and in the dentist group (1.5:1 and 1.7:1, respectively), whereas in PTs the ratio is 1:1. More males with mild disability are found in the FMs group, with a female:male (F:M) ratio of 0.7.

The F:M ratio is more pronounced in the groups with higher neck related disability. For moderate disability, the ratio is 7:1 in controls, 2.2:1 in PTs, FMs had 13 females and 0 males, while dentists had male predominance of 0.44 (F:M). A severe form of disability was not found in the control population, whereas 7 medical professionals reported this disability, representing 1.5% of all NDI questionnaires (6 women and 1 man).

When analysing the gender distribution of the different levels of disability using the x^2^ test for trend, a female predominance was found in the control group and in the group of PTs (*p* = 0.005 and *p* = 0.01, respectively), while no significant difference was found in FMs and dentists (*p* = 0.81 and *p* = 0.32, respectively). 

In contrast to the gender distribution, the age distribution showed that age increases with the degree of disability in all studied groups except dentists (Table 2). The test for a linear trend showed that the control population had an average increase of 11.19 ± 1.9 years (*p* < 0.001) across all disability categories, followed by FMs with 10.9 ± 1.9 (*p* < 0.001) and PTs with 4.8 ± 1 years increase (*p* = 0.01). Dentists had an increase of 2.4 ± 1.7 years, but with a statistically non-significant *p* = 0.14. (Table 2).

**Figure 2:**
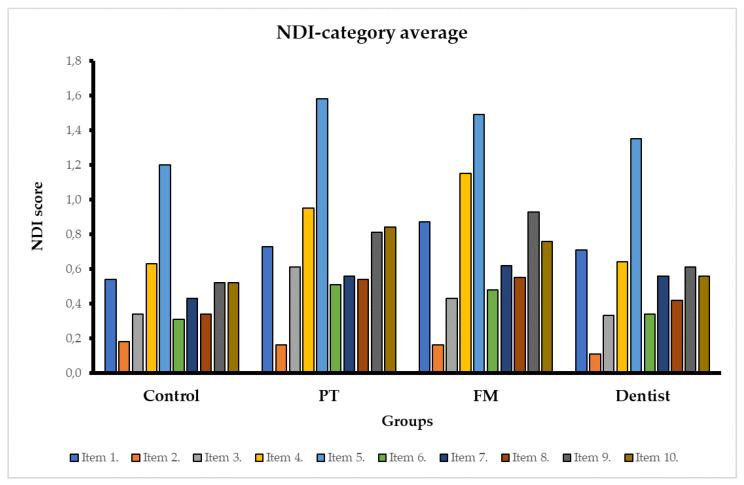
The average score of NDI categories in different medical professions. 1. Neck pain severity, 2. Self-care, 3. Lifting, 4. Reading, 5. Occurrence of headache, 6. Concentration, 7. Driving, 8. Sleeping, 9. Occupational activity, 10. Recreational activity.

The average score of the different categories of the NDI questionnaire is shown in Figure 2.

The results of the question on headache (item 5) had an average score of 1.2 or more in all groups and contributed most to the overall result. Headache occurred in 73% of controls and in more than 80% of healthcare professionals. The second highest item was the question on reading (item 4), which had an average score of more than 0.6 in all groups. According to the NDI, another peak score was observed in sleeping and recreation items (items 9 and 10) in the PTs and FMs, which was above 0.76, in contrast to the dentists and the control group.

Comparison of average NDI item scores between groups by multiple linear regression showed that PTs had higher values of NDI score for problems with lifting things (item 3) when compared to controls.

NDI scores for the severity of neck pain (item 1), reading problems (item 4), the occurrence of headaches (item 5), concentration problems (item 6), difficulties in performing occupational (item 9), and recreational activities (item 10) were higher in PTs and FMs compared to the average NDI scores of corresponding items in controls. The difference was also statistically significant.

No statistically significant differences were found between groups in NDI scores for difficulty with self-care (item 2), driving (item 7), and sleeping (item 8).

Finally, the dentist group did not show a statistically significant difference from the control group in any of the NDI categories. Matrices for multiple linear regression can be provided upon request.

## 4. Discussion

This is the first paper to describe the discrepancy between the prevalence of neck pain and the degree of neck-related disability resulting from WMSDs. The presence of a painful condition as an entity does not imply the presence of disability, which ultimately limits the quality of the health professional’s professional and social life. Examining the functionality of the individual using the NDI questionnaire contributes to the understanding of neck pain in the context of disability.

According to our data, the point prevalence of neck pain is higher in all medical professionals than in the control population, so we would expect higher levels of disability in all medical professionals. This was refuted by the results of our study. For example, looking only at the prevalence of neck pain, dentists could be considered the second most affected professional group after FMs, despite being the youngest of all groups. The prevalence of neck pain among dentists in this study (50.4%) was similar to that reported by Leggat and Smith (57%) and Prudhvi et al. (56%) [19,26]. However, when analysing the NDI% results, we found that this population did not differ from controls in either mean NDI score or degree of disability.

Multiple linear regression analyses confirmed no differences in NDI score between dentists and controls for all ten categories of the NDI (1. Neck pain severity, 2. Self-care, 3. Lifting, 4. Reading, 5. Occurrence of headache, 6. Concentration, 7. Driving, 8. Sleeping, 9. Occupational activity, 10. Recreational activity).

This leads us to conclusion that this group has high functional capacity and the lowest disability, although one in two dentists reported neck pain in the last year. This may be the result of improving working posture by using an ergonomic dental chair with arm support, using lightweight instruments with better handle design, using prismatic glasses, chair-side stretches [27,28,29], or implementing ergonomic training courses during dental high-school and college [17,30,31,32].

Gender or age had no effect on dentists’ NDI scores in our study. In contrast to our results, Gandolfi et al. concluded that WMSDs in dentists increase with age, with a peak between 36 and 50 years of age, and Sustová et al. reported a statistically significant effect of age on the occurrence of moderate and major problems in WMSDs in dentists [14,33].

Female gender has been associated with a higher prevalence of neck pain in dentists in many papers [33,34,35,36], but we found no difference between genders in either the prevalence of neck pain or the absolute NDI score.

PTs are occupational groups that perform the most physically demanding work of all the groups studied. Lifting and transferring patients, working in sometimes uncomfortable postures, high number of patients, and repetitive tasks are everyday problems in this occupational group and may contribute to the development of WMSDs. This type of physical work can only be compared to the work of nurses caring for bedridden patients. Both, low back pain as well as pain in the neck and shoulders are common in this profession. Low back pain in PTs has been associated with younger physical therapists and their inexperience with correct postures or the need to prove themselves in the profession [10,37]. Increased use of force during professional activity combined with overload in work environments is opposite to the principles of pain prevention and treatment they prescribe to their patients [38,39]. It has been reported that women are more likely to suffer from neck and shoulder pain, which was also found in our study, partly due to the lower strength when lifting and transferring patients [3,9,10].

This was confirmed by multiple linear regression by finding that only PTs have significantly higher scores in the NDI category of lifting compared to controls.

One of the reasons for this is the lack of education on proper lifting and transferring of patients as described by Fan et al. in 2022. who reported that only 5 of 224 physical therapists surveyed had received formal ergonomics training [40]. Dentists, on the other hand, have mandatory ergonomics courses. In addition, there is evidence that PTs tend not to report WMSDs or seek professional help relying only on their own expertise, and sometimes working through pain, thus exacerbating their condition [41].

On the other hand, FMs have the most sedentary occupation of all health professionals included in this study. They formed the oldest group. Their disability level was surprisingly similar to that of PTs with a high neck-related disability level. These data were in contrast to those reported in the literature that only 20% of FMs had WMSDs [21,22]. Multiple linear regression revealed that both PTs and FMs had significantly higher scores on neck pain severity (item 1), reading problems (item 4), the occurrence of headaches (item 5), concentration problems (item 6), difficulties in performing occupational (item 9), and recreational activities (item 10).

Although FMs did not show gender differences in disability levels, similarities with FMs can be drawn through worsening of neck disability level with age. PTs reach a higher level of disability more quickly in about five years, whereas this period is more than ten years for FMs. In the previous section, we described possible reasons for neck disability in PTs, but in FMs, we believe that other pathophysiological mechanisms may be responsible. FMs spend most of the workday at a computer, and during this time the neck spends more time in an immobile working position. This may result in pain in the facet joints due to decreased secretion of synovial fluid or gradual loss of water in the intervertebral discs due to increased pressure from the dynamic vertebral segment, as previously described [1]. Regular breaks between patients with simple neck range of motion exercises could slow down this process. Males and females are equally affected in this group.

We hypothesise that both excessive isotonic muscle activity (PTs) and isometric activity of the paravertebral neck muscles (FMs) could lead to persistent muscle spasm after the workday. This can lead to headaches, reading difficulties, and less leisure or occupational activity than the individual would like.

By isolating at-risk healthcare professionals (PTs with five years of work experience, especially female, and FMs with 10 years of work experience), we have the ability to intervene. Education about WMSD and advice on prevention or treatment should be integrated into lifelong learning through regular lectures and workshops. Preventive actions, like kinesitherapy for health professionals, should be considered by the institution where they work or by a specific medical association. The importance of exercise has already been reported in physical therapists: those who exercised less than 4 h per week were more likely to have musculoskeletal complaints than those who exercised more than 4 h [40].

Neck related problems are usually not severe enough to affect NDI scores for the self-care (item 2), driving (item 7), and sleeping (item 8) categories, and they do not differ from those of controls in any group.

### 4.1. Limitations of the Study

Although a strong correlation between the NDI and the depression index PHQ-9 has been previously described [24], the NDI is known for disregarding psychological and emotional factors, thus partly limiting interpretation of the results. A combination of functional and psychological indices is recommended for future studies on WMSDs.

Regarding age distribution, PTs and control participants were age-matched, whereas FMs were older and dentists were younger than control participants, which may partly explain the better results among dentists and the worse ones among FMs.

Some data from the study participants and the control population regarding duration of working years, daily working hours, physical activity levels, and comorbidities were not included. Further studies should include these data for more detailed analyses.

### 4.2. Conclusions

It would be less accurate to assess WMSDs based on prevalence alone. It is more appropriate to use functional indices for each region studied. For example, if only the prevalence of neck pain is considered, dentists could be considered the second most affected occupational group after FMs, despite being the youngest of all groups. However, when analysing the NDI% results, we found that this population had high functional capacity and the lowest disability from neck pain, comparable to the control population. In addition, gender or age had no effect on NDI scores in this population.

FMs, who were the oldest group, had the highest percentage of disability. Neck symptoms were related to age, with older participants tending to be represented in the higher disability groups, with no association with gender.

PTs were the second most affected group, with females predominating in all disability categories and PTs tending to be five years older as disability increased.

To conclude, by isolating at-risk healthcare professionals (PTs with five years of work experience, especially female, and FMs with 10 years of work experience), we have the ability to intervene. Education about WMSD and advice on prevention or treatment should be integrated into lifelong learning through regular lectures and workshops. Preventive actions, like kinesitherapy for health professionals, should be considered by the institution where they work or by a specific medical association.

## Figures and Tables

**Table 1:** Baseline characteristics of participants in neck pain study in medical professionals.

		Participants			
		FM (n = 103)	Dentist (n = 113)	PT (n = 239)	Control (n = 112)
Age, years *p*-value *	Median (IQR)	49 (36–59) 0.002 * (FM vs. C)	42 (30.5–48.5) 0.002 * (D vs. C)	43 (34–54) 0.07 (PT vs. C)	43 (29–58) N/A
Gender	M/F (%) M/F (n)	18.5/81.5 19/84	37.2/62.8 42/71	25.5/74.5 61/178	50.9/49.1 57/55

Abbreviations: FM—family medicine group, PT—physiotherapist group, C—control group, D—dentist, M—males, F—females; * Kolmogorov–Smirnov test was used for age comparison.

**Table 2:** Age and gender distribution at different levels of disability as determined by the Neck Disability Index.

	Control	PT	FM	Dentist	Control		PT		FM		Dentist		Control	PT	FM	Dentist
					F	M	F	M	F	M	F	M				
NDI% Disability	Percentage and Number of Participants n% (n)				GENDER n% (n)								AGE (Mean ± SD)			
No (0–8)	55.4 (62)	41 (98)	42.7 (44)	54.9 (62)	41.8 (23)	68 (39)	37.7 (67)	50.8 (31)	42.9 (36)	42.1 (8)	49.3 (35)	64.3 (27)	38.4 (13)	40.2 (12)	38.4 (14)	40.0 (10)
Mild (10–28)	37.5 (42)	47.3 (113)	43.7 (45)	38.1 (43)	45.5 (25)	29.8 (17)	48.3 (86)	44.2 (27)	41.7 (35)	52.6 (10)	45.1 (32)	26.2 (11)	50.4 (14)	44.2 (11)	50.4 (14)	40.5 (12)
Moderate (30–48)	7.1 (8)	9.6 (23)	12.6 (13)	6.2 (7)	12.7 (7)	1.8 (1)	11.2 (20)	4.9 (3)	15.5 (13)	0	4.2 (3)	9.5 (4)	59.4 (10)	49.3 (9)	59.4 (10)	48.9 (8)
Severe (50–68)	0	2.1 (5)	1 (1)	0.8 (1)	0	0	2.8 (5)	0	0	5.3 (1)	1.4 (1)	0	0	58.6 (3)	N/A	N/A
Total	100 (112)	100 (239)	100 (103)	100 (113)	100 (55)	100 (57)	100 (178)	100 (61)	100 (84)	100 (19)	100 (71)	100 (42)	44.4 (15)	43.3 (12)	44.4 (15)	40.8 (10)

Abbreviations: FM—family medicine group, PT—physiotherapist group, C—control group, D—dentist, M—males, F—females, NDI—Neck Disability Index, N/A not applicable.

## Data Availability

Matrices for multiple linear regression can be provided upon request from the corresponding author. The data are not publicly available due to privacy issues.

## Supplementary Materials

The following supporting information can be downloaded at: https://www.mdpi.com/article/10.3390/healthcare11040581/s1. Figure S1. Flowchart of the study. FMs—family medicine specialists, PTs—physiotherapists, control—control population, dentists. Total number representing approximate number of medical professionals in Croatia in the last five years.

## References

[1] Halkai K.R., Halkai R.S., Sulgante S., Sanadi R.M., Ara S.A., Zainab H., Kuriadom S.T., Munaga S., Chitumalla R. (2022). Work-related musculoskeletal disorders among dentists and their prevention through ergonomic interventions—A systematic review. Int. J. Occup. Saf. Health.

[2] Milhem M., Kalichman L., Ezra D., Alperovitch-Najenson D. (2016). Work-related musculoskeletal disorders among physical therapists: A comprehensive narrative review. Int. J. Occup. Med. Environ. Health.

[3] Rozenfeld V., Ribak J., Danziger J., Tsamir J., Carmeli E. (2010). Prevalence, risk factors and preventive strategies in work-related musculoskeletal disorders among Israeli physical therapists. Physiother. Res. Int..

[4] Rugelj D. (2003). Low back pain and other work-related musculoskeletal problems among physiotherapists. Appl. Ergon..

[5] Salik Y., Özcan A. (2004). Work-related musculoskeletal disorders: A survey of physical therapists in Izmir-Turkey. BMC Musculoskelet. Disord..

[6] West D.J., Gardner D. (2001). Occupational injuries of physiotherapists in North and Central Queensland. Aust. J. Physiother..

[7] Adegoke B.O., Akodu A.K., Oyeyemi A.L. (2008). Work-related musculoskeletal disorders among Nigerian Physiotherapists. BMC Musculoskelet. Disord..

[8] Alperovitch-Najenson D., Treger I., Kalichman L. (2014). Physical Therapists Versus Nurses in a Rehabilitation Hospital: Comparing Prevalence of Work-Related Musculoskeletal Complaints and Working Conditions. Arch. Environ. Occup. Healh.

[9] Alrowayeh H.N., Alshatti T.A., Aljadi S.H., Fares M., Alshamire M.M., Alwazan S.S. (2010). Prevalence, characteristics, and impacts of work-related musculoskeletal disorders: A survey among physical therapists in the State of Kuwait. BMC Musculoskelet. Disord..

[10] Bork B.E., Cook T.M., Rosecrance J.C., Engelhardt K.A., Thomason M.E.J., Wauford I.J., Worley R.K. (1996). Work-related musculoskeletal disorders among physical therapists. Phys. Ther..

[11] Cromie J.E., Robertson V.J., Best M.O. (2000). Work-Related Musculoskeletal Disorders in Physical Therapists: Prevalence, Severity, Risks, and Responses. Phys. Ther..

[12] Glover W., McGregor A., Sullivan C., Hague J. (2005). Work-related musculoskeletal disorders affecting members of the Chartered Society of Physiotherapy. Physiotherapy.

[13] Aljanakh M., Shaikh S., Siddiqui A., Al-Mansour M., Hassan S.S. (2015). Prevalence of musculoskeletal disorders among dentists in the Ha’il Region of Saudi Arabia. Ann. Saudi Med..

[14] Gandolfi M., Zamparini F., Spinelli A., Risi A., Prati C. (2021). Musculoskeletal Disorders among Italian Dentists and Dental Hygienists. Int. J. Environ. Res. Public Health.

[15] Ísper Garbin A.J., Barreto Soares G., Moreira Arcieri R., Adas Saliba Garbin C., Siqueira C.E. (2017). Musculoskeletal disorders and perception of working conditions: A survey of Brazilian dentists in São Paulo. Int. J. Occup. Med. Environ. Health.

[16] Jaoude S.B., Naaman N., Nehme E., Gebeily J., Daou M. (2017). Work-Related musculoskeletal pain among lebanese dentists: An epidemiological study. Niger. J. Clin. Pract..

[17] Koni A., Kufersin M., Ronchese F., Travan M., Cadenaro M., Larese Filon F. (2018). Approach to prevention of musculoskeletal symptoms in dental students: An interventional study. Med. Lav..

[18] Kumar S., Baliga M.R., Kumar V. (2013). Prevalence of work-related musculoskeletal complaints among dentists in India: A national cross-sectional survey. Indian J. Dent. Res..

[19] Prudhvi K., Murthy K.R.V. (2016). Self-reported musculoskeletal pain among dentists in Visakhapatnam: A 12-month prevalence study. Indian J. Dent. Res..

[20] Vodanović M., Sović S., Galić I. (2016). Occupational Health Problems among Dentists in Croatia. Acta Stomatol. Croat..

[21] Rambabu T., Suneetha K. (2014). Prevalence of work related musculoskeletal disorders among physicians, surgeons and dentists: A comparative study. Ann. Med Health Sci. Res..

[22] Kitzmann A.S., Fethke N.B., Baratz K.H., Zimmerman M.B., Hackbarth D.J., Gehrs K.M. (2012). A Survey Study of Musculoskeletal Disorders Among Eye Care Physicians Compared with Family Medicine Physicians. Ophthalmology.

[23] Vernon H., Mior S. (1991). The Neck Disability Index: A study of reliability and validity. J. Manip. Physiol. Ther..

[24] Aljinović J., Barun B., Poljičanin A., Marinović I., Vlak T., Pivalica D., Benzon B. (2022). Croatian version of the neck disability index can distinguish between acute, chronic and no neck pain: Results of a validation study. Wien. Klin. Wochenschr..

[25] Pleho D., Hadžiomerović A.M., Pleho K., Pleho J., Remić D., Arslanagić D., Lazić M., Alibegović A. (2021). Work Caused Musculoskeletal Disorders in Health Professionals. J. Health Sci..

[26] Leggat P.A., Smith D.R. (2006). Musculoskeletal disorders self-reported by dentists in Queensland, Australia. Aust. Dent. J..

[27] Hallaj S., Razi S. (2016). Design and Evaluation of an Arm Support for Prevention of MSDs in Dentists. Advances in Ergonomics in Design: Proceedings of the AHFE 2016 International Conference on Ergonomics in Design, Orlando, FL, USA, 27–31 July 2016.

[28] Katano K., Nakajima K., Saito M., Kawano Y., Takeda T., Fukuda K. (2021). Effects of Line of Vision on Posture, Muscle Activity and Sitting Balance During Tooth Preparation. Int. Dent. J..

[29] Rempel D., Lee D.L., Dawson K., Loomer P. (2012). The effects of periodontal curette handle weight and diameter on arm pain: A four-month randomized controlled trial. J. Am. Dent. Assoc..

[30] Dabaghi-Tabriz F., Bahramian A., Rahbar M., Esmailzadeh M., Alami H. (2020). Ergonomic Evaluation of Senior Undergraduate Students and Effect of Instruction Regarding Ergonomic Principles on It. Maedica.

[31] Movahhed T., Dehghani M., Arghami S., Arghami A. (2016). Do dental students have a neutral working posture?. J. Back Musculoskelet. Rehabil..

[32] Peros K., Vodanovic M., Mestrovic S., Rosin-Grget K., Valic M. (2011). Physical Fitness Course in the Dental Curriculum and Prevention of Low Back Pain. J. Dent. Educ..

[33] Sustová Z., Hodacová L., Kapitán M. (2013). The prevalence of musculoskeletal disorders among dentists in the Czech Republic. Acta Med..

[34] Puriene A., Aleksejuniene J., Petrauskiene J., Balciuniene I., Janulyte V. (2008). Self-reported occupational health issues among Lithuanian dentists. Ind. Health.

[35] Sharma P., Golchha V. (2011). Awareness among Indian dentist regarding the role of physical activity in prevention of work related musculoskeletal disorders. Indian J. Dent. Res..

[36] Valachi B. (2008). Musculoskeletal health of the woman dentist: Distinctive interventions for a growing population. J. Calif. Dent. Assoc..

[37] Molumphy M., Unger B., Jensen G.M., Lopopolo R.B. (1985). Incidence of Work-Related Low Back Pain in Physical Therapists. Phys. Ther..

[38] Alghadir A., Zafar H., Iqbal Z.A., Al-Eisa E. (2017). Work-Related Low Back Pain Among Physical Therapists in Riyadh, Saudi Arabia. Work. Health Saf..

[39] Iqbal Z., Alghadir A. (2015). Prevalence of work-related musculoskeletal disorders among physical therapists. Med. Pr..

[40] Fan L.J., Liu S., Jin T., Gan J.G., Wang F.Y., Wang H.T., Lin T. (2022). Ergonomic risk factors and work-related musculoskeletal disorders in clinical physiotherapy. Front. Public Health.

[41] Campo M., Weiser S., Koenig K.L., Nordin M. (2008). Work-Related Musculoskeletal Disorders in Physical Therapists: A Prospective Cohort Study With 1-Year Follow-up. Phys. Ther..

