# Supplementary material for: Neck Disability Index Detects Higher Neck-Related Disability Levels among Physiotherapists and Family Medicine Specialists than among Dentists
Source: Healthcare (Basel). 2023 Feb 15;11(4):581. doi: 10.3390/healthcare11040581 (PMC9957489; doi:10.3390/healthcare11040581)
Supplement: Supplementary file 1 [file healthcare-11-00581-s001.zip › healthcare-2158284-Supplementary.pdf]

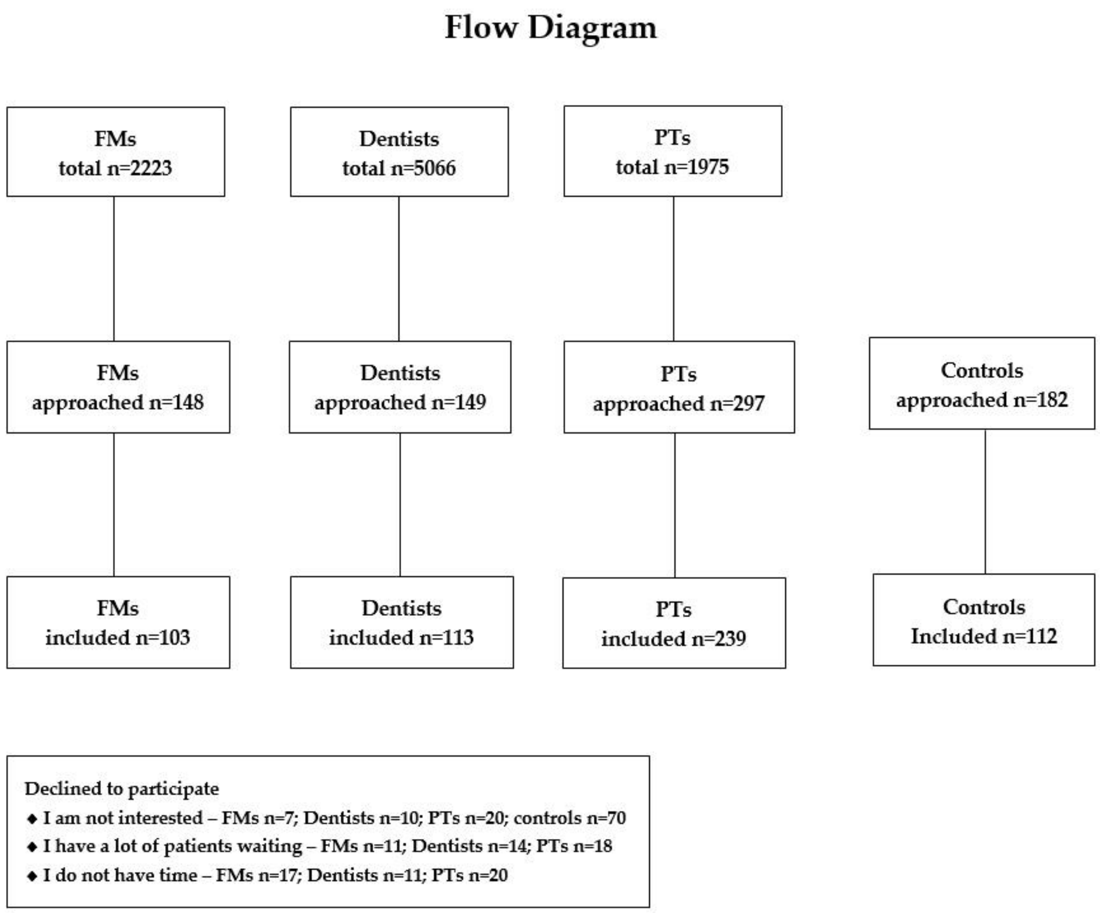

Figure S1. Flowchart of the study. FMs-family medicine specialists, PTs-physiotherapists, control-control population, dentists. Total number representing approximate number of medical professionals in Croatia in the last five years
